# Measuring response to treatment in axial spondyloarthritis using quantitative imaging biomarkers: a prospective observational cohort study

**DOI:** 10.1259/bjr.20220530

**Published:** 2023-10-25

**Authors:** Alexis Jones, Timothy JP Bray, Naomi S Sakai, Alan JP Bainbridge, Coziana Ciurtin, Margaret A Hall-Craggs

**Affiliations:** 1 Department of Rheumatology, University College London Hospitals NHS Foundation Trust, London, UK; 2 Centre for Medical Imaging, University College London, London, UK; 3 Department of Medical Physics and Biomedical Engineering, University College London Hospital, London, UK

## Abstract

**Objective:**

Objective assessments of disease activity and response to treatment in axial spondyloarthritis (axSpA) remain a challenge; quantitative imaging biomarkers (QIBs) of inflammation could enhance assessments of disease activity and therapeutic response. We aimed to determine the responsiveness of QIBs obtained from diffusion-weighted imaging (DW-MRI) and chemical shift-encoded MRI (CSE-MRI) using the partially automated Bone Edema and Adiposity Characterisation with Histograms (BEACH) software tool in axSpA patients undergoing biologic therapy.

**Methods:**

We conducted a prospective longitudinal cohort study, including 30 patients with axSpA undergoing biologic therapy. Patients were scanned before and after biologic therapy using conventional MRI, DWI and CSE-MRI at 3T. Apparent diffusion coefficient (ADC) and proton density fat fraction (PDFF) were assessed using the BEACH tool (https://github.com/TJPBray/BEACH), and conventional MR images were assessed using established visual scoring methods by expert radiologists. Responsiveness – the ability of the MRI measurements to capture changes in disease occurring as a result of biologic therapy – was assessed using the standardized response mean (SRM). Inter-reader reliability of the ADC and PDFF maps was assessed using Bland-Altman limits of agreement analysis and the intraclass correlation coefficient.

**Results:**

Responsiveness to therapy was moderate for ADC-based parameters (SRM 0.50) and comparable to established visual scoring methods for bone marrow oedema (SRM 0.53). Interobserver variability was lower for QIBs compared with conventional visual scores methods.

**Conclusions:**

QIBs measured using the BEACH tool are sensitive to changes in inflammation in axSpA following biologic therapy, with similar responsiveness and lower interobserver variability to visual scoring by expert radiologists.

**Advances in knowledge:**

QIBs measured using the partially automated BEACH tool offer an objective measure of response to biologic therapy in axSpA.

## Introduction

Accurate assessments of disease activity are imperative for the initiation and continuation of therapies and play a vital role in the assessment of novel drugs in clinical trials. Current disease activity scores in axial spondyloarthritis are reliant on patient reported outcome measures (PROMS) of salient symptoms. The Bath Ankylosing Spondylitis Disease Activity Index (BASDAI) is the most utilised score, aggregating patient measures of fatigue, back pain, joint pain and/or swelling, enthesitis, intensity and duration of morning stiffness into a single value.^1^ The Ankylosing Spondylitis Disease Activity Score (ASDAS) combines elements of the BASDAI and patient global assessment with a laboratory measure of inflammation, either C-reactive protein (CRP) or erythrocyte sedimentation rate (ESR).^2^ These scores have been validated and show significant response to biologic therapy in a number of clinical trials.^3,4^ A number of other PROMS are available for axSpA, including those assessing functional limitation with axSpA (the Bath Ankylosing Spondylitis Functional Index and the Health assessment questionnaire-Spondyloarthropathy)^5^ as well as scores incorporating measures of spinal mobility (the Bath Ankylosing Spondylitis Metrology Index).^6^ Whilst these scores reflect important disease characteristics, their interpretation is difficult in a number of clinical scenarios. Firstly, there is a high incidence of chronic pain and fibromyalgia in patients with axial spondyloarthritis^7^ confounding measures of pain, stiffness, fatigue and function. For this reason, patients with spondyloarthritis and fibromyalgia are often excluded from clinical drug trials, although their presence in the clinical setting is arguably high. The incidence of mechanical spinal issues in the general population is high,^8^ and many symptoms mimic those of spondyloarthritis. Following the initiation of any new drug, a percentage of patients will experience symptoms attributed to placebo rather than the drug itself. Thus, there is a growing need for robust and objective assessments of inflammation in axSpA, which do not rely on subjective reporting of symptoms. MRI of the spine and sacroiliac joints (SIJs) is increasingly used for this purpose.

The Spondyloarthritis Research Consortium of Canada (SPARCC) has developed a scoring system for bone marrow oedema (SPARCC BME)^9^ and an SIJ structural score (SPARCC SSS) for fatty change, erosions and ankylosis,^10^ allowing for a quantitative assessment of disease activity in the SIJs. The SPARCC BME scoring system has been shown to predict remission with biologic therapy in clinical trials.^11,12^ The SPARCC score, however, is labour-intensive, expertise-dependent and not applicable to routine clinical practice. The subjective nature of SPARCC scoring can also create variability between observers, reducing the responsiveness of the measure. In clinical trials, this could lead to a reduction in effect size and a subsequent reduction in power.

Recent studies have shown promise for the use of quantitative imaging biomarkers (QIBs) in the assessment of disease activity in spondyloarthritis.^13–15^ Diffusion-weighted imaging (DWI) has emerged as a quantitative method for assessment of bone marrow oedema.^16^ The apparent diffusion coefficient (ADC) measured by DWI reflects the diffusion of free fluids within extracellular spaces and is increased in areas of bone marrow oedema, thought to reflect expansion of the extracellular space by free water. Increased ADC values in the SIJs of both adult axSpA and adolescents with enthesitis-related arthritis have been reported and show response to treatment.^17^ Chemical shift-encoded MRI (CSE-MRI) measures the proton density fat fraction (PDFF) in the marrow, which is sensitive to the presence of bone marrow oedema (since oedema reduces the normal marrow fat) and marrow fat deposition (also known as fat metaplasia).^18^ Fat metaplasia in the SIJ is thought to be a post-inflammatory phenomenon and may reflect overall the burden of structural damage. It has been shown to predict spinal radiographic progression in axSpA^19^

**Figure 1.**
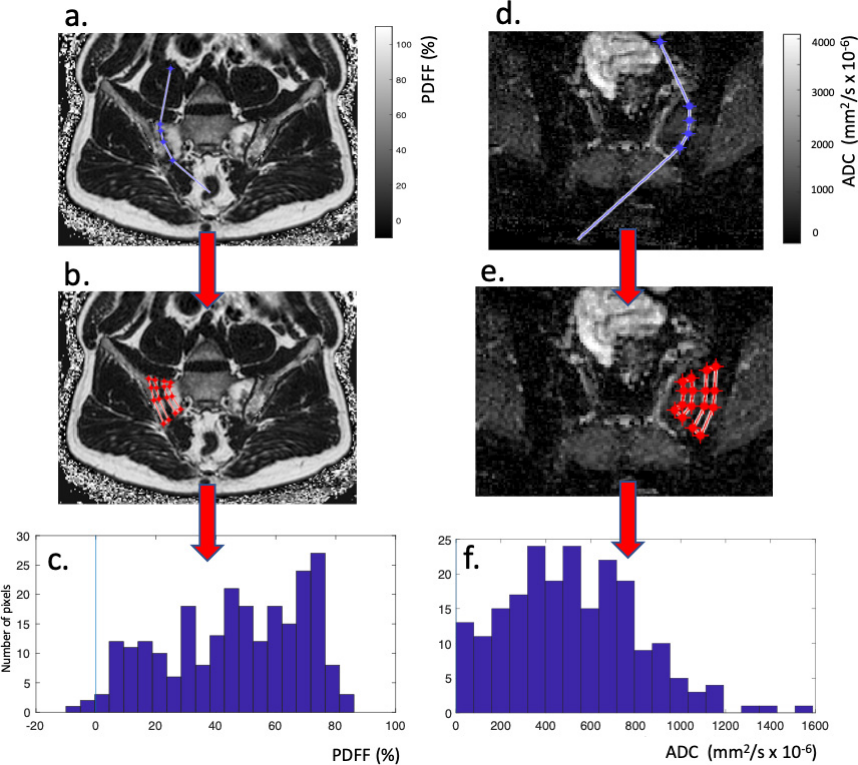
Partially-automated image analysis using the BEACH tool. The observer is prompted to define the line of the sacroiliac joint (**a,d**), using anchor lines to define the angle between the joint and bone cortex. The software automatically propagates polygonal ROIs onto subchondral bone (**b,e**) and histograms are generated from the defined ROI (**c,f**). The complete process has been previously described in.^20^

At present, assessment of ADC and fat metaplasia requires a skilled reader to manually plot regions of interest (ROIs). This is a time-consuming, specialised, skill which demonstrates variability amongst reporters. To address this, Bray and colleagues have developed the partially automated Bone Edema and Adiposity Characterisation with Histograms (BEACH) tool for ADC and PDFF measurement in subchondral bone marrow.^8^ This tool requires only that the observer defines the line of the sacroiliac joint and then automatically propagates ROIs onto subchondral bone, thus reducing the degree of subjectivity in the interpretation (Figure 1). The tool uses histographic analysis to ‘target’ the areas of maximal abnormality within the ROI. By only requiring the user to define the joint, this method requires less user expertise than producing a conventional ROI, potentially offering greater objectivity and precision and also saving time.

In this study, we aimed to test the hypothesis that ADC- and PDFF-based histographic parameters derived from the BEACH tool are valid and responsive markers of response to biologic therapy, based on MRI and clinical assessments at baseline (prior to starting biologic treatment) and at 12–16 weeks after starting biologic treatment (according to the type of biologic used) in a cohort of 30 patients with spondyloarthritis. Responsiveness was assessed in terms of standardised response means (SRMs) for the various QIBs and was compared against the SRM for visual SPARCC scoring. Validity was assessed by the correlation of QIBs with conventional MRI and clinical activity scores. We also assessed whether QIBs at baseline were able to predict clinical response determined by linear regression.

## Methods

This study received ethical approval from the London Riverside Ethics Committee (IRAS 208355).

### Subjects

Subjects were recruited prospectively from **** between April 2018 and July 2019. Patients aged 18 to 85 years with a diagnosis of axSpA according to 2009 ASAS criteria^21^ and active disease according to the National Institute of Clinical Excellence (NICE guidelines NG65) criteria were approached to take part. Exclusion criteria included contraindications to MRI such as metallic implants, pacemaker, severe claustrophobia, pregnancy, body weight > 150 kg. Previous treatment with an oral, intra-articular or intramuscular glucocorticoid within 4 weeks prior to inclusion was not allowed. Patients continued in the study if their MRI fulfiled ASAS criteria for sacroiliitis,^22^ and they were eligible for their first biologic drug (biologic naive) or a change biologic therapy (switchers) in accordance with best practice (NICE guidelines NG65). A repeat scan was performed after 12 weeks ( ± 2 weeks) of continuous anti-TNF treatment or 16 weeks ( ± 2 weeks) of anti-IL 17 treatment. Patients were withdrawn from the study if biologic therapy was declined by the patient, contraindicated or stopped owing to adverse events before weeks 12–16. All patients provided written informed consent.

### Clinical assessments

Information regarding patient demographics (age, sex and ethnicity), body mass index, disease duration, history of peripheral arthritis and enthesitis, extra-articular manifestations, human leukocyte antigen (HLA) B27 status, drug history and smoking history were recorded at baseline. In addition, patients were assessed for fibromyalgia/chronic pain in accordance with ACR criteria.^23^ Clinical examination including tender and swollen joint count and assessment of peripheral enthesitis at baseline and after 12–16 weeks of treatment. BASDAI and ASDAS scores as well as CRP and ESR were recorded at baseline and after 12–16 weeks of continuous treatment. A clinical response was assessed on the basis of a BASDAI improvement of ≥1.2 and an improvement in spinal VAS of ≥1. This criterion is in accordance with NICE criteria to reflect real-life clinical practice. Other clinical response measures included a reduction in BASDAI by 50% (BASDAI 50), a clinical important improvement in ASDAS (CII ASDAS) defined as a change in ASDAS > 1.1 and inactive disease defined as an ASDAS of <1.3 (ASDAS ID).

### MRI acquisition

All quantitative and conventional MRI scans of the SIJs and lumbar spine were performed on a 3.0T Ingenia scanner (Philips, Amsterdam, Netherlands), in a single attendance. Quantitative imaging consisted of (1) chemical shift-encoded MRI (CSE-MRI), also known as Dixon MRI, PDFF maps and (2) DWI, producing ADC maps. CSE-MRI was performed using the methodology previously described.^18,20^ Briefly, the images were acquired using a multiecho gradient echo acquisition with bipolar readouts (first echo time 1.17 ms, echo spacing 1.6 ms, flip angle 3°, repetition time 25 ms, matrix size 320 × 320, pixel spacing 1.76 × 1.76 mm); fat water separation was performed using an investigational version of the Philips mDixon Quant software, assuming 10-peak model of human adipose tissue and a single R_2_* decay term for the bone marrow.^18^ Diffusion-weighted imaging was performed with b-values of 0, 50 and 600 s/mm^2^ using a standard Stejskal-Tanner sequence with spectrally attenuated inversion recovery (SPAIR) fat suppression and echo-planar readout. The DWI acquisition was optimised to minimise fat-ghosting artifacts. Conventional MRI consisted of *T*_2_-weighted short tau inversion recovery (STIR), *T*_1_-weighted turbo spin echo and fat-suppressed *T*_1_-weighted turbo spin echo post-contrast imaging. All conventional MRI images of the sacroiliac joints were acquired in both angled coronal (parallel to the sacrum) and angled transverse (perpendicular to the sacrum) planes. Post-contrast scans were also acquired through the thoracolumbar spine.

### Quantitative image analysis

QIB measurements were obtained from the PDFF and ADC maps using the BEACH tool, as shown in Figure 1 and as described in detail by Bray et al..^20^ The software for this tool is publicly available at https://github.com/TJPBray/BEACH. The BEACH tool incorporates two main elements: (1) partially automated definition of regions-of-interest (ROIs) and (2) analysis of pixel values within the ROI using histographic analysis. The assessor is prompted to define the line of the sacroiliac joint using a single series of connected straight lines (an open polygon). Anchor lines are used to define the angle made by the joint, enabling the shape of the polygonal ROIs to be tailored to the precise geometry of the subchondral bone in each patient. The software then automatically generates a pair of polygonal ROIs in the subchondral bone, to a depth of 10 mm, on either side of the joint. The procedure is repeated for both the left and right sacroiliac joints, on each slice, until the subchondral bone included in the imaging volume had been fully sampled. In the case of ADC maps, we included all slices where the synovial joint was visible, whereas alternate slices were used for the PDFF maps due to the much thinner slices (2 mm) available from CSE-MRI. To be consistent with the visual scoring systems used for comparison in this work, only the subchondral bone corresponding to the synovial part of the joint was defined (the bone corresponding to the ligamentous part of the joint was excluded). For each patient, pixel values from the total volume of defined subchondral bone (*i.e.,* from all ROIs) were analysed histographically. The ROIs for the BEACH tool were generated by two radiology registrars with four and six years of experience in musculoskeletal MRI, respectively, and experience of using this tool in previous studies.

For both ADC and PDFF, the 25^th^, 50^th^, 75^th^ and 90^th^ percentiles of the distribution were measured, referred to as ADC_25_, ADC_median_ ADC_75_ and ADC_90_ and PDFF_25_, PDFF_median_, PDFF_75_ and PDFF_90_ for ADC and PDFF, respectively. Mean ADC and mean PDFF were also recorded. Examples of ADC and PDFF histograms generated using the BEACH tool and corresponding percentile measurements can be found in seen in Figure 1.

### Qualitative image scoring

Each set of conventional MR images (including STIR, *T*_1_-weighted and contrast-enhanced images) were assessed by two radiologists with over 6 and 25 years of experience in musculoskeletal radiology, who scored the images independently using the SPARCC system. Images were read on a dedicated research workstation where the reader was blinded to clinical diagnosis, treatment and quantitative measurements. The presence of bone marrow oedema (BME) was evaluated in six consecutive slices based on SIJs divided into eight quadrants. Each quadrant was scored for the presence/absence of BME (*i.e.,* one or 0). An additional score of 1 was added if the BME in a quadrant was more than 10 mm deep, and another score of 1 was added if the BME in a quadrant was at least as intense as the cerebrospinal fluid. A total score out of 72 was reached for SPARCC BME. In addition, the presence of fatty change was assessed using the SPARCC SIJ structural score (SPARCC SSS). The presence/absence of fatty lesions per quadrant was calculated. A total score out of 50 was obtained.

### Statistical analysis

To assess responsiveness, QIB measurements and SPARCC scores were first averaged over the two readers. First, a paired student’s t test was performed to compare BEACH-derived parameters before and after biologic therapy. Responsiveness was then calculated using the standardised response mean (SRM), calculated as the mean change score for each BEACH parameter divided by the standard deviation of the corresponding change score. The SRM values were defined as small (0.2–0.5), moderate (0.5–0.8) or large (>0.8). The relationship between ADC and PDFF QIBs, SPARCC scores and clinical scores was assessed using Pearson’s r correlation coefficient. Binary logistic regression was used to investigate the association between clinical outcomes (dependent variables) and baseline ADC and change in ADC scores (independent variables). Inter-reader reliability of the ADC and PDFF maps was assessed using Bland-Altman limits of agreement analysis.

## Results

Patient demographics are shown in Table 1. Thirty-one patients consented to take part in the study. One patient was withdrawn owing to side effects of the biologic treatment before week 6 (*n* = 30). The ratio of females to males was 16:14. The mean age was 42.7 years. 13 patients had ankylosing spondylitis (AS) and 17 non-radiographic axial spondyloarthritis (nr-axSpA). Average disease duration was 7.5 years. 26.7% of patients had peripheral arthritis and 16.7% peripheral enthesitis. 60% of patients were HLA B27 positive. Nine patients (30%) were also diagnosed with fibromyalgia. Biologic treatment initiated included Humira, Etanercept biosimilar (Benepali) and Secukinumab. 25 patients were biologic naive and 5 patients were switched to either a second (*n* = 4) or third (*n* = 1) biologic therapy owing to primary or secondary failure.

**Table 1.** Baseline characteristic of study participants

Baseline characteristics	Number/percentage
Patient number	30
Age (mean years)	42.7 (22-67)
Females: Males	16:14
Ankylosing spondylitis: non-radiographic axSpA	13:17
Mean duration from symptom onset to diagnosis (years)	7.5 (SD 5.1)
Mean duration of symptoms (years)	14.3 (SD 11.1)
Peripheral arthritis	8 (26.7%)
Peripheral enthesitis	5 (16.7%)
HLA B27	18 (60.0%)
Fibromyalgia	9 (30.0%)
Biologic naive	25 (83.3%)
second biologic therapy	4 (13.3%)
third biologic therapy	1 (3.3%)
Baseline SPARCC (BMO)	15.3 (SD 15.2)
Baselines SPARCC SSS (fat)	17.7 (SD 8.1)
Baseline SPARCC SSS (erosion)	25.3 (SD 4.3)
Baseline SPARCC SSS (ankylosis)	2 (SD 0.4)

BMO, bone marrow oedema;HLA, Human leucocyte antigen SD, standard deviation; SPARCC, Spondyloarthritis Research Consortium of Canada.

### Responsiveness

**Figure 2.**
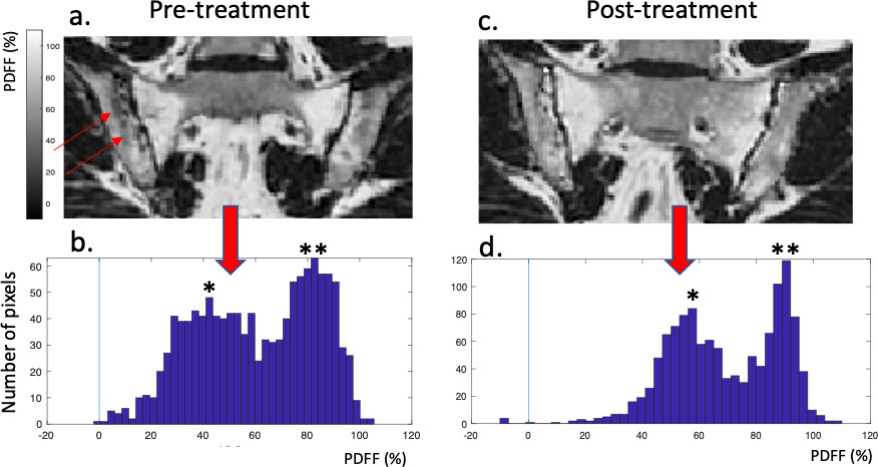
Changes in histograms with treatment. Pre-treatment (**a**) and post-treatment (**c**) images and corresponding histograms (**b, d**) are shown. In this subject, the histogram has two peaks (denoted * and **) which may correspond to oedema and normal marrow/fat metaplasia, respectively. On the post-treatment scan, there is a rightward shift in the histogram with both peaks moving upwards in terms of PDFF, although the lower ‘oedema’ peak remains present.

Pairwise comparisons showed significant reductions after treatment for both ADC median (*p* = 0.012) and SPARCC BME scores (*p* = 0.008), in addition to clinical parameters including BASDAI (p = <0.001), spinal VAS (p = <0.001), ASDAS CRP (p = <0.001) and ASDAS ESR (p = <0.001) (Table 2). Both ADC median and SPARCC BME showed moderate responsiveness following biologic therapy (SRM 0.52 and 0.50, respectively) (Table 3). There was no significant difference in SPARCC SSS (fat) scores and PDFF scores before and after treatment. PDFF-based QIBSs showed small responsiveness to biologic therapy. An example of PDFF histograms before and after treatment is shown in Figure 2.

**Table 2.** Standardised response means for clinical, SPARCC and QIBs scores

	Pre-biologics (mean)	Post-biologics (mean)	*p* value	Confidence interval (CI)	Standardised response mean
BASDAI	6.88	4.91	<0.001^a^	2.97–1.15	0.89
Spinal VAS	6.90	4.76	<0.001^a^	3.07–1.19	0.85
ASDAS CRP	3.32	2.4	<0.001^a^	1.32–−0.53	0.88
ASDAS ESR	3.19	2.29	<0.001^a^	1.24 -–0.56	0.98
CRP	5.35	1.99	0.020	6.134 to −0.5798	0.45
SPARCC BME	15.18	10.6	0.008^a^	7.850 to −1.317	0.52
SPARCC SSS (fat)	17.73	19.63	0.161	0.7982 to 4.598	0.26
ADC mean	291.22	277.16	0.056	28.49–0.37	0.37
ADC median	195.21	170.52	0.012a	43.53–5.83	0.50
ADC 25	8.59	4.09	0.159	10.83–1.87	0.27
ADC 75	476.4	457.2	0.078	40.62–2.35	0.34
ADC 90	585.3	573.9	0.170	46.31–8.56	0.26
PDFF mean	57.39	59.54	0.098	0.426–4.73	0.31
PDFF median	56.79	58.76	0.161	0.83–4.78	0.27
PDFF 25	47.84	50.26	0.127	0.73–5.57	0.29
PDFF 75	66.84	68.92	0.100	0.43–4.57	0.31
PDFF 90	51.21	50.53	0.132	0.54–3.90	0.28

ADC, apparent diffusion coefficient; BASDAI, Bath ankylosing Spondylitis Disease Activity Index; BME, Bone marrow oedema; CRP, c reactive protein; PDFF, proton density fat fraction; SPARCC, Spondyloarthritis Research Consortium of Canada; SSS, sacroiliac joint structural score; VAS, visual analogue score.

a denotes *p* value < 0.05. Shaded boxes represent standardised response means > 0.40;

**Table 3.** Correlation between SPARCC and QIB scores at baseline using Pearson’s r correlation coefficient

QIB	SPARCC BME	SPARCC SSS (fat)
ADC mean	0.67	0.11
ADC median	0.57	0.13
ADC 25	0.32	0.21
ADC 75	0.70	0.06
ADC 90	0.68	0.08
PDFF mean	0.27	0.52
PDFF median	0.17	0.50
PDFF 25	0.37	0.42
PDFF 75	0.06	0.59
PDFF 90	0.09	0.52
PDFF 75	0.14	0.59
PDFF 90	0.12	0.60

ADC, apparent diffusion coefficient; PDFF, proton density fat fraction.; QIB, quantitative imaging biomarker.

Shaded boxes represent correlation coefficients >0.50.

### ADC and PDFF correlations with qualitative MRI and clinical scores

Correlations of ADC- and PDFF-based BEACH parameters with SPARCC BME and SPARCC SSS (fat) scores are shown in Table 3. At baseline, ADC mean, ADC median, ADC 75 and ADC 90 correlated with SPARCC BME and PDFF mean, median and PDFF 75 and PDFF 90 correlated with SPARCC SSS. There was an inverse correlation between the change in PDFF mean and median before and after treatment with the change in SPARCC BME before and after treatment.

ADC- and PDFF-based parameters did not significantly correlate with BASDAI, spinal VAS, ASDAS ESR, ASDAS CRP or laboratory variables (CRP or ESR). There was no significant difference in QIB scores between clinical responders or non-clinical responders and QIBS could not predict those patients who reached BASDAI 50, CII ASDAS or ASDAS ID.

### Inter-observer variability

**Figure 3.**
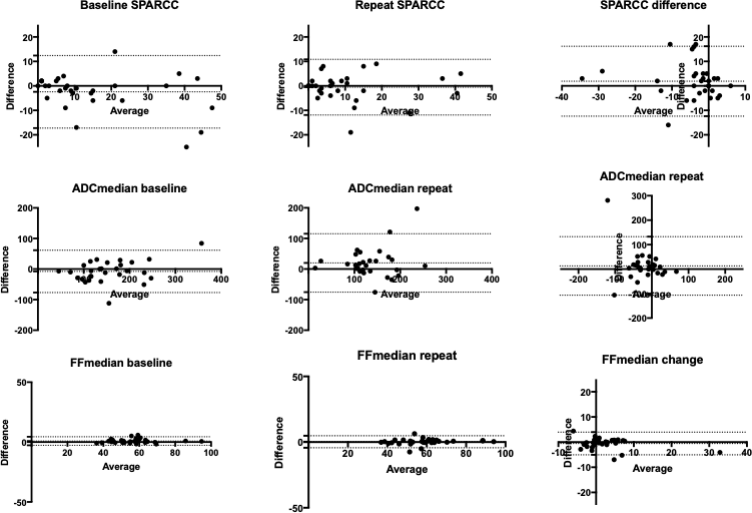
Bland-Altman plots for interobserver variability. Bland-Altman plots with 95% limits of agreement are shown for SPARCC scores (top row), for ADC_median_ (middle row) and FF_median_ (bottom row). The three columns show plots for pre-treatment measurements, post-treatment measurements and difference between pre- and post treatment measurements, (from left to right).

Bland-Altman plots for inter-reader reliability for selected BEACH parameters are shown in Figure 3. Bias (LoA) was: −2.43 (-17.3 to 12.4) for baseline SPARCC, −0.53 (-11.9 to 10.9) for repeat SPARCC, 1.9 (-12.4 to 16.2) for SPARCC change, −7.7 (-77 to 61) for baseline ADC median, 19.8 (-76 to 115) for repeat ADC median, 13 (-107 to 133) for ADC median change, 0.85% (-2.7 to 4.4) for FF median baseline, 0.24% (-43 to 4.8) for FF median repeat and −0.55% (-5.1 to 4.0) for PDFF median change. Assessment of intraobserver agreement using the BEACH tool has previously been assessed by the authors and results have shown excellent agreement^20^

## Discussion

There has been growing interest in using quantitative imaging methods as an objective means to assess inflammation of the sacroiliac joints. However, most studies have used simple manual delineation to derive ADC measurements, which introduces unwanted subjectivity into the assessment, contrary to the aims of quantitative imaging. In contrast, the BEACH tool provides a partially automated ROI placement and histographic analysis, which is less expertise-dependent and therefore has the potential to improve the consistency of disease assessment in clinical practice.

In this study, we showed that parameters derived using the BEACH tool are valid and responsive measures of response to biologic therapy. Of the parameters evaluated, the ADC median of the SIJ joints was particularly sensitive to change and demonstrated a similar responsiveness to SPARCC BME following biologic therapy.

Although previous studies have shown that oedema can affect PDFF measurements (through loss of the normal marrow fat), PDFF values and SPARCC SSS (fat) scores did not show significant change before and after treatment with biologic therapy and demonstrated non-significant SRMs. Other studies have shown similar results for quantitative structural scores.^24^ One explanation could be that structural changes in bone marrow take longer than 12–16 weeks to be detected by MRI. PDFF scores correlated well with SPARCC SSS (fat) scores at baseline, arguing for their validity as a tool for measuring of fat fraction. It would be of value to assess changes in PDFF over a longer period to determine whether this could be used to assess the gradual development of structural damage (easily missed by visual assessment alone).

The lack of correlation between QIB scores and clinical parameters may be explained by the fact that our imaging scores assessed inflammation in the sacroiliac joints only, excluding any contribution from spinal inflammation, peripheral joint, entheseal pain and fatigue captured in clinical scores. Fatigue, in particular, is a complex symptom which does not always parallel objective reductions in inflammation in rheumatic disease.^25^ The heterogeneity of our study population may have also contributed to this. We deliberately included patients with concomitant fibromyalgia and those patients switching biologic therapy following primary or secondary failure to reflect real-life clinical practice. Notably, only 27% of our patients achieved a BASDAI 50 response at 12 weeks compared with 50–60% of biologic naïve patients typically reported in clinical trials.^26^ This result could not be attributed to a higher BMI in our patient cohort.

A significant advantage of our study was its single centre prospective design. All patients were imaged using the same MRI system and the same protocol defined at predefined time points. Variations in MRI scanner, sequences and timings were, therefore, minimized. Few prospective studies have used ADC measurements to assess responses to biologic treatment in axSpA and in these studies ROIs were used.^27^ This study validates the use of the BEACH tool as a semi-automated method of calculating QIBS which is faster, requires less expertise and also demonstrates high inter-rater reliability.

The study was designed as a methodological study and has a small study population (*n* = 30), limiting its power. This has been offset in part by the matched pairs study design. Further work should include a larger prospective study to determine the effect of age, sex, disease duration and concomitant conditions such as fibromyalgia on the use of this tool in the assessment of disease activity and response to treatment.

The purpose of introducing a new metric to assess disease activity in axSpA is not to replace clinical disease activity scores but to assist clinicians in those cases where patient reported outcome measures prove difficult to interpret. This includes patients with fibromyalgia, chronic pain, long-term mechanical damage from ankylosing spondylitis or concomitant degenerative spinal changes. A new measure of inflammation (and particularly changes with treatment) could also assist in the setting of clinical trials, where placebo may affect a significant proportion of patients.

### Conclusion

QIBs measured using the BEACH tool are sensitive to changes in inflammation in axSpA following biologic therapy, with similar responsiveness and lower interobserver variability to visual scoring by expert radiologists.
